# Curriculum Innovation: Implementing Anti-Racism Education in Residency Training: A Multicenter Pilot Study

**DOI:** 10.1212/NE9.0000000000200288

**Published:** 2026-01-08

**Authors:** Alissa A. Thomas, Rebecca Pollard, Robin Ulep, Nimish A. Mohile

**Affiliations:** 1 Department of Neurological Sciences, University of Vermont Larner College of Medicine, Burlington;; 2 Department of Neurology, University of Pittsburgh Medical Center, PA;; 3 Department of Neurology, Icahn School of Medicine at Mount Sinai, New York, NY; and; 4 Department of Neurology, University of Rochester Medical Center, NY.

## Abstract

**Background and Objectives:**

In March 2022, the American Academy of Neurology (AAN) published “The AAN Anti-Racism Education Program,” an online curriculum designed to educate neurologists to recognize anti-racism as a professional competency, identify systemic and institutional racism and inequities, and apply an equity framework and related skills to patient care and health care systems. In the 2022–2023 academic year, a working group of neurology residency programs piloted the incorporation of this program into their didactic curriculum. The aim of this study was to determine the feasibility and impact of implementing the AAN Anti-Racism Education Program into neurology residency education.

**Methods:**

This was a multicenter feasibility pilot study of neurology residency programs that participated voluntarily. A representative resident and faculty leader from each program met monthly with the AAN Anti-Racism Curriculum Chair, completed facilitator training, and incorporated the curriculum into their academic programming. The amount of protected time, as well as schedule and format for content delivery and discussion, was at the discretion of each program. Participating faculty and residents were invited to complete an anonymous IRB-approved REDCap survey at the completion of the curriculum.

**Results:**

Sixteen adult neurology residency programs participated in the pilot study. At the end of the academic year, 58 participants responded to the survey, including 18 faculty members and 40 residents. Of those who responded, 86% reported working through the online modules, generally spending 30–120 minutes per module. Most respondents reported that they would recommend this program to other residents and residency programs (84.5%), and most believed that residency training should include education about racism (91.4%). Respondents reported that, after completing this curriculum, they were more confident working with people from different racial backgrounds (77.6%), more confident caring for patients from different racial backgrounds (75.5%), more comfortable talking about racism (82.5%), better able to understand the impact of race on medical care and health outcomes (85.9%), and more confident that they would react or intervene to promote anti-racism (79.3%).

**Discussion:**

Implementation of the AAN Anti-Racism Education Program was feasible in a variety of programs and resulted in improved confidence among residents and faculty in addressing issues around race and racism.

## Introduction

Neurology residency programs are tasked with teaching residents to understand the structural and social determinants of health of the populations they serve.^1^ To accomplish this, programs must implement curricula to eliminate health disparities and to address patients' needs. Residents are evaluated on their ability to understand health systems and demonstrate knowledge of community health needs and disparities, with an ultimate goal of leading innovations to improve health disparities.^2^ Individual residency programs may meet program requirements in a way that fits the needs of their patient population, local and regional communities, and training program. Many neurology residency programs look to the American Academy of Neurology (AAN) for guidance on educational content to ensure alignment with the American Council of Graduate Medical Education (ACGME) core competencies and milestones necessary to practice neurology.

In alignment with its mission to “promote brain health for all” and vision “to be indispensable to its members,” in March 2022, the AAN developed an Anti-Racism Education Program aimed at providing learning opportunities, specifically to neurologists, to better understand inequities in our society that can be barriers to health equity and brain health for all.^3^ Before this program, there was no formal curriculum designed specifically for neurologists to learn about health disparities related to race and racism. Other anti-racism curricula have been developed for residents and undergraduates with successful implementation and lessons learned.^4-6^ Because neurology residents are responsible for caring for a diverse group of patients, education about health inequities is an important part of their training.

### Objectives

The objective of this study was to determine the feasibility of implementing the AAN Anti-Racism Education Program into standard neurology resident educational curricula. The primary aim was to incorporate this innovative course into residency training. A secondary aim was to assess the impact of the curriculum on residents' comfort level with health disparities and racism.

## Methods

### Curriculum Content

The AAN Anti-Racism Education Program is a web-based, self-paced, on-demand course for neurologists, consisting of 4 online modules that include case studies and vignettes, member experiences, self-reflection, videos, and additional tools and resources for self-study^3^:

1. Module 1: Setting the stage—reflections on race, identity, and socialization
2. Module 2: The history of racism in neurology—member experiences with bias and racism
3. Module 3: Patient care stories—vignettes in clinical and academic neurology
4. Module 4: Institutional structure—racism in training and anti-racism leadership

The course was designed to be completed in 16 hours over the course of several months. The AAN Anti-Racism Education Program is published on the AAN website and is free to all AAN members.

### Program Selection

The Anti-Racism Education Program was introduced at the 2022 AAN Annual Meeting, where neurology residents and program directors were in attendance, after which a small working group was formed to participate in a joint effort to incorporate the Anti-Racism Education Program into their training programs the following academic year. Additional programs were invited through word-of-mouth and through the AAN Program Director Synapse. Each participating program selected a representative resident leader and faculty sponsor (program director, associate program director, or other faculty member).

### Curriculum Implementation

This pilot study focused on incorporating the existing AAN Anti-Racism Education Program into neurology residency programs' didactic curriculum. All residents who volunteered to participate in the program were AAN members through their sponsoring institution, and thus, the curriculum was free to access. The resident and faculty leaders from each program met on monthly video conference calls to plan the pilot study, build comfort and confidence with the materials of the curriculum, troubleshoot challenges, and receive training from members of the AAN Anti-Racism Curriculum Working Group on how to be an effective group facilitator. The working group recommended that residency programs have residents work through the online modules individually and then participate in facilitated group discussions after each module. Programs were encouraged to incorporate these facilitated discussions into their regular academic sessions, with a suggestion of spacing out the 4 modules quarterly throughout the year. Programs could decide whether to offer facilitated discussion as part of a noon conference series, an academic half-day, or a separate session. Programs also decided how much, if any, protected time to provide the residents for completion of the modules and participation in the facilitated discussions.

### Facilitator Training

For programs that included group discussion sessions, resident and faculty leaders received training for the facilitator role. The training included introducing the curriculum, setting the tone for safe discussion, creating a safe space and brave space, managing complex discussions, and encouraging engagement from all participants. Facilitators were given guidance on establishing ground rules, building connections, incorporating observation and role playing, navigating conflict, validating and facilitating discussion of feelings, and responding to emotions with curious questions.^7^ Facilitator training also included hypothetical challenging scenarios and possible solutions.

### Assessment and Feedback

Residents and program faculty (program directors, associate program directors, or other participating faculty members) were sent an anonymous feedback survey at the end of the one-year pilot project. The survey was reviewed and approved by the University of Vermont Institutional Review Board as an Education Research study, and the study was granted a waiver of informed consent documentation. Responses were collected and analyzed using REDCap.

### Standard Protocol Approvals, Registrations, and Participant Consents

The survey was approved by the Institutional Review Board at the University of Vermont, with a waiver of informed consent. All responses were entered anonymously through a secure REDCap database.

### Data Availability

Anonymized data not published within this article will be made available by request from any qualified investigator.

## Results

**Figure 1:**
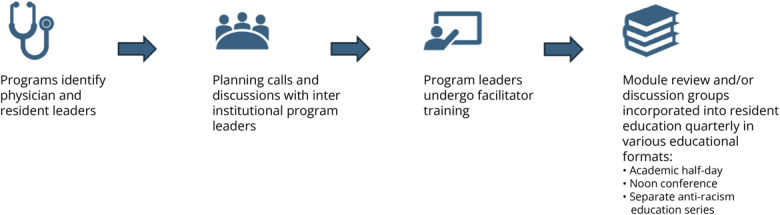
Schema Demonstrating the Steps and Time Line for Program Participation and Curriculum Implementation

Sixteen adult neurology residency programs participated in the pilot study. Programs were from across the United States, including the Northeast, Southeast, Midwest, South Central, and Pacific. The programs were all at academic medical centers and included a range of small and large programs. There was a mix of implementation methods, with format and scheduling at the discretion of each individual program, as shown in Figure 1.

### Participation and Survey Response

Surveys were sent to participating residents and faculty. The survey response rate was 19%, with 58 responses, including 40 from residents and 18 from faculty. The survey response rate was 51.4% for faculty and 14.5% for residents. Residents were at the PGY2-5 levels of training.

### Feasibility and Implementation

**Figure 2:**
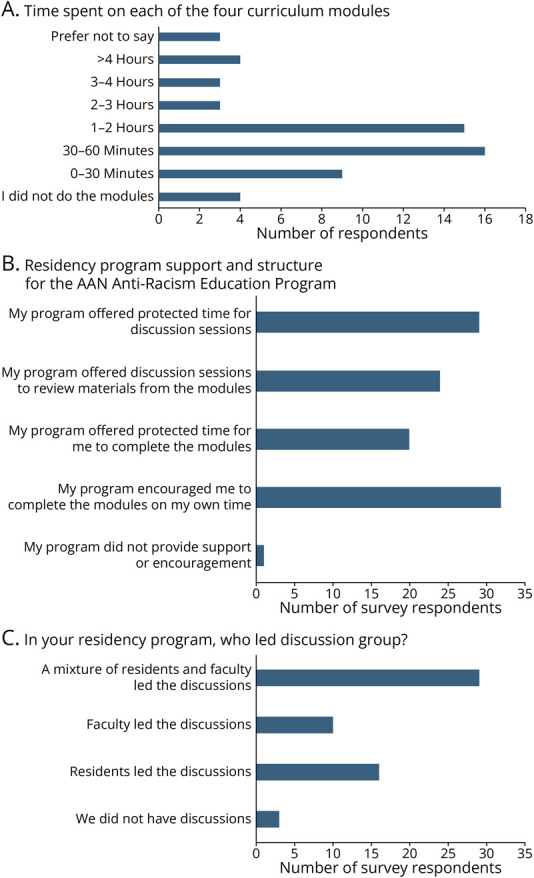
Survey Responses to How Residency Programs Implemented the AAN Anti-Racism Program (A) The time residents and faculty spent working through the 4 online modules. (B) How programs supported the residents in working through the modules and discussing the material in discussion groups. (C) The distribution of program leaders who facilitated the discussions. AAN = American Academy of Neurology.

Among respondents (residents and faculty), 86% reported that they worked through at least some of the online curriculum modules, and more than half of the participants worked through all 4 curriculum modules. Time spent on each module ranged from <30 minutes to >4 hours (Figure 2A). Over a third of respondents (34.5%) reported that they had protected time to complete the online modules, while half of the respondents (50%) reported that they had protected time to complete the group discussion sessions (Figure 2B). Most discussion sessions (50%) were led by a faculty and resident pair, while 28% were led by a resident alone and 17% by a faculty member alone (Figure 2C). Forty-one percent of respondents reported that their program offered discussion sessions as part of the curriculum work.

### Feedback and Engagement

To assess the impact of the curriculum, the survey included a series of statements and a 5-point Likert scale that ranged from strongly agree to strongly disagree with each statement. The survey data are provided in the Table. Most of the survey respondents (76%) agreed or strongly agreed that the AAN Anti-Racism Education Program was a worthwhile use of their time while 2% disagreed. More than a third (36%) of participants reported that the AAN curriculum was novel, and that they had not previously received any anti-racism training or education. Participation in the pilot project increased confidence in working with people from different backgrounds for 79% of participants, and it increased comfort with talking about racism for 84% of participants. Most participants (79%) felt more confident that they would react or intervene to promote anti-racism, were they to witness racism. Eighty-six percent of survey respondents would recommend the curriculum to other residents and residency programs. Nearly all participants (91.4%) believed that residency programs should include education about racism.

**Table:** Engagement and Impact of the AAN Anti-Racism Education Program in Neurology Education

	Strongly agree (%)	Agree (%)	Neither agree nor disagree (%)	Disagree (%)	Strongly disagree (%)	Prefer not to say (%)
I had previously received anti-racism training or education outside of the AAN curriculum	15.5	32.8	15.5	24.1	12.1	0
I am more confident working with people who come from different racial backgrounds	29.3	48.3	19	1.7	1.7	0
I am more confident in my ability to care for patients from different racial backgrounds	28.1	47.4	21.1	3.5	0	0
I feel more comfortable talking about racism	31.6	50.9	14	1.8	1.8	0
I can better understand how race impacts medical care and health outcomes	33.3	52.6	10.5	3.5	0	0
I feel more comfortable talking with my patients about race	15.8	43.9	35.1	5.3	0	0
I feel more comfortable talking with other health care professionals about racism	31	53.4	10.3	5.2	0	0
If I witness racism, I am more confident that I would react or intervene to promote anti-racism	25.9	53.4	17.2	3.4	0	0
I believe that residency programs should include education about racism	62.1	29.3	5.2	3.4	0	0
I am able to recognize racism when I see or hear it	35.1	561	7	1.8	0	0
I would benefit from further education about racism	46.6	37.9	12.1	3.4	0	0
I would recommend this program to other residents and residency programs	48.3	36.2	10.3	3.4	0	0

Abbreviation: AAN = American Academy of Neurology.

Survey respondents were asked to rate each of the following statements on a Likert scale ranging from strongly agree to strongly disagree. The survey questions and responses are included in the table.

## Discussion

Neurology residents are responsible for the care of patients who present with a wide spectrum of neurologic symptoms, inclusive of patients of all ages, sexes, races, socioeconomic statuses, backgrounds, abilities, and levels of health literacy. As part of their training, residents need to learn not only the pathophysiology, diagnosis, and management of neurologic diseases but also how to navigate a complex health care system and understand the factors contributing to health disparities and inequities. Among these factors, an understanding of the impact of race and racism is essential in providing equitable neurologic care. The AAN Anti-Racism Education Program provides such training that is relevant to neurologists.

This pilot study demonstrates that the AAN Anti-Racism Curriculum can be practically incorporated into the existing educational programming of neurology residency programs. For programs that can incorporate anti-racism education into their standing curriculum, this experience demonstrates a number of feasible models for implementation, including the use of protected time for completion of the modules, facilitator training for sensitive topics, and facilitated discussion with resident and/or faculty leads.

Most of the residents and program directors who participated in this pilot believe that anti-racism education should be a mandatory part of neurology training programs. Residents are charged with caring for all patients who enter their health care system, treating all patients with respect and dignity, and providing the highest quality care. The framework of the AAN Anti-Racism Education Program gives space for self-reflection on one's own understanding of identity and implicit biases that an individual may carry. These concepts may be applied more broadly to the care of all patients.

There were several challenges encountered with implementing the curriculum and participating in this pilot. Because the pilot was intended to demonstrate the feasibility of a new curriculum, there was no standardized method of delivery as each program selected different logistical models and amounts of protected time, thus introducing variability in models of implementation and levels of participation. Furthermore, the voluntary nature of the 16 participating programs suggests that these programs were already considering the value of this form of education, thus biasing the supportive response. Within each program, there was also variability in how strictly the modules and discussion sessions were enforced among residents; as such, not all residents in all participating programs fully completed the course by the end of the year.

In addition, there are several limitations to the qualitative nature of our evaluation metrics. The 14.5% response rate of residents may bias our data toward residents who might have been more engaged in this project, and our study could not effectively capture those who were disengaged or disinterested in this type of education. Nonetheless, the survey results suggest that the curriculum achieved its goal of increasing the comfort level and confidence of participants in working with a racially diverse population of patients and coworkers and their ability to communicate about racism and race-related health disparities. We do not know how many residents from each participating program completed the survey because we intentionally did not collect any institution-level demographic data. As such, the positive results related may be more limited to a smaller number of programs and may not reflect the experience of participants at all 16 locations. Because of the anonymous nature of our feedback survey, we do not know the implementation design across individual institutions to be able to report which model was most popular or most successful. Narrative feedback suggested that many residents would find value in revisiting these educational materials again during their training.

Notably, at the time that this pilot study was implemented, education related to Diversity, Equity and Inclusion (DEI) was encouraged in many academic institutions. Many institutional DEI education programs have since changed, dismantled, or shifted focus because of new federal regulations put in place in 2025, which may present new challenges to expanding this program to the 155 ACGME-accredited adult neurology residency programs. However, the online work-at-your-own-pace nature of the AAN Anti-Racism Curriculum will remain an option for residents and residency programs who would like to have access to these materials, either for programmatic curriculum or for individual learning on a voluntary basis.

This study offers groundwork that can guide future directions at broadening residency training regarding race and anti-racism. This experience adds to previously published frameworks that provide tips for implementing and teaching anti-racism curriculum in medical education^8^ and models for anti-racism teaching for residents.^9^ As noted in this previous work, racism is ubiquitous in medicine, and the success of curriculum interventions requires multilayered, longitudinal approaches that are woven into the residency curriculum.^9^ Program leaders from undergraduate and graduate medical education have recognized the importance of intentional training and institutional support for anti-racism education.^10^ Important future steps include evaluating the efficacy of different models of content delivery, assessing the impact of the curriculum based on participants' demographic backgrounds, encouraging greater support at the institutional level and across more programs, addressing challenges with implementation, and continually improving the Anti-Racism Education Program in light of a shifting cultural climate. Implementation of the AAN Anti-Racism Education Program into neurology residency training was feasible and resulted in increased confidence among residents and faculty in their approach to race and racism in clinical practice.

## Glossary

AAN: American Academy of Neurology
ACGME: American Council of Graduate Medical Education
DEI: Diversity, Equity and Inclusion
